# Knowledge, attitudes, and perception toward new psychoactive substances among Saudi university students: a cross-sectional study

**DOI:** 10.3389/fpsyt.2026.1877711

**Published:** 2026-07-08

**Authors:** Abdullah Al Hamid, Abdulrahman A. Alnijadi, Lujain Alrubaia, Lina Albraik, Mousa Alfaifi

**Affiliations:** Department of Pharmacy Practice, College of Clinical Pharmacy, King Faisal University, Al-Ahsa, Saudi Arabia

**Keywords:** new psychoactive substances, NPS, Saudi Arabia, substance use, university students

## Abstract

**Background:**

New psychoactive substances (NPS) are defined as chemical compounds that are synthesized or extracted from natural sources with the aim of producing psychoactive effects similar to those produced by controlled substances but not regulated under current legislation. Worldwide, the rise in the use of NPS has been outpacing regulation and education efforts, leading to substantial health risks, especially for the young adults’ population. In Saudi Arabia, substance-use prevention is supported through the Saudi 2030 initiative led by the Ministry of Interior and National Committee for Narcotics Control; nonetheless, most studies conducted at the national level have centered on traditional drugs only.

**Objectives:**

The purpose of this research was to evaluate the knowledge, attitude, and perception related to NPS among King Faisal University students, and to determine the predictors of better knowledge and permissive attitude.

**Methods:**

A cross-sectional study was conducted with 300 students via an anonymous web-based survey questionnaire to assess sociodemographic characteristics and knowledge, attitude, and perception measures. Ethical approval was obtained from King Faisal University (reference no. KFU-REC-2026-MAR-ETHICS4051) on the 12^th^ of March 2025. Chi-squared test/Fisher’s exact test were applied to understand the relationship between sociodemographic variables and level of knowledge, and independent samples t-test/ANOVA for comparison of mean attitude scores. Logistic regression analysis was conducted to identify independent predictors of high knowledge and permissive beliefs.

**Results:**

Out of the respondents (74% females, mean age 18–24 years), (58%) had high levels of knowledge regarding NPS. Access to NPS was easy for (57%), mostly through online media (70.8%) and past usage of NPS was admitted by (39.7%). Significantly greater knowledge regarding NPS was shown by females compared to males (64.0% vs 41.0%; P<0.001), while being male predicted poor knowledge (AOR = 0.407, 95% CI:0.229–0.722, P = 0.002). Prior NPS use (AOR = 2.502, 95% CI:1.209–5.176, P = 0.013) and easy access (AOR = 2.298, 95% CI:1.233–4.285, P = 0.009) independently predicted permissive beliefs. Common use motivations included social gatherings (47.9%), weight loss (40.3%), and stress reduction (37.0%).

**Conclusions:**

Students have moderate to high levels of knowledge, their attitudes towards NPS were significantly influenced by ease of access on the Internet and previous usage experience. This indicates the necessity of educational measures to tackle the increasing threat posed by NPS to Saudi universities.

## 1 Introduction

The global picture regarding the illicit drug trade has changed drastically owing to the quick entry and variation of New Psychoactive Substances (NPS) (1). The United Nations Office on Drugs and Crime (UNODC) defines NPS as “any substance of abuse, either in a pure form or as a preparation, that is not regulated under the 1961 Single Convention on Narcotic Drugs or the 1971 Convention on Psychotropic Substances but may pose risks to public health (2).” NPS are intentionally developed with the aim to achieve similar outcomes as the common illicit drugs but at the same time bypass any legal regulations through modifications in the drugs’ chemical structure (3). Thus, NPs comprise a diverse group of drugs of different pharmacological activities, sources and formulation (4). Common NPS classes reported include synthetic cannabinoids, synthetic cathinones, novel opioids, phenethylamines, and dissociative agents. These substances exert their effects through different modes of action, such as stimulation of cannabinoid receptors, enhancement of monoamine neurotransmission, activation of opioid receptors, or antagonism of N-methyl-D-aspartate (NMDA) receptors. Compared with traditional illicit drugs, many NPS exhibit unpredictable potency and pharmacological profiles, increasing the risk of adverse events. Commonly reported toxicities of NPS included agitation, psychosis, seizures, cardiovascular complications, respiratory depression, and death. The rapid emergence of new compounds and the limited awareness of their effects pose significant challenges for public health systems and highlight the importance of improving knowledge and risk awareness among young adults, particularly university students.

A significant part of the NPS landscape is the phenomenon of “pharming”, the use of prescription and over-the-counter medications for psychoactive effects (5). This includes the non-medical use of medications such as antihistamines, cough medicines, decongestants and prescription drugs, often obtained without proper medical supervision and with substantial health risks (6, 7). Pharming is an alternative route of NPS use, particularly traditional NPS use, especially in young populations who may consider these products to be safer alternatives to illicit drugs (8). Particularly concerning are the mental health consequences of NPS use, which have been consistently associated with psychosis, suicidality and other serious psychiatric outcomes (9, 10).

Maintenance of health for young Saudis is another vital objective under the purview of the Saudi Vision 2030 framework launched by the Saudi government and that focuses on enhancing the “Quality of Life” while avoiding the danger of substance use (11). In Saudi Arabia, public health concern around substance misuse has mainly focused on traditionally controlled substances such as crystal meth/Shabu (12). However, NPS present an additional challenge because they are chemically diverse, rapidly changing, and may be less familiar than conventional drugs to young people. Previously, local literature on substance use has focused mostly on conventional drugs, implying a lack of knowledge about the dangers of these “new” chemicals among Saudi youth (13).

The efficiency of public health interventions is highly reliant on the level of awareness and attitudes (14). However, global studies reveal a persistent gap in the required information to detect the risks of NPS such as psychosis, heart problems, and fatality (15, 16). Thus, the aim of this study was to evaluate the knowledge, attitude, and perception related to NPS among King Faisal University students, Saudi Arabia. Also, to determine the predictors of better knowledge and permissive attitude towards NPS use. A permissive attitude towards NPS refers to a tendency to regard their use as acceptable or low risk resulting in greater willingness to experiment or approve their use (17). To the best of our knowledge, this study is among the few conducted in Saudi Arabia investigating the connection between students’ perceptions and the use of the NPS (18–20). This provides valuable information for university management and policymakers to design specific educational materials.

## 2 Methods

### 2.1 Study design

A cross-sectional descriptive survey design in assessing the Knowledge, Attitudes, and Perceptions of students towards NPS. The data were gathered using an online survey designed using Google Forms.

### 2.2 Online survey administration and response handling

The questionnaire was distributed electronically through university email and WhatsApp student communication groups. The survey was administered using Google Forms with the “Limit to 1 response” setting enabled, requiring respondents to sign in and restricting responses to one submission per Google account. All survey items were mandatory; therefore, no missing item-level data were generated, and only complete questionnaires were included in the final analysis. Because the survey link was disseminated broadly through email and WhatsApp rather than through a controlled sampling frame, the exact number of students who received or viewed the invitation could not be determined, and response/view rates could not be calculated. The final analysis included 300 complete responses.

### 2.3 Respondents and data collection

The sample consisted of students enrolled in various programs at King Faisal University located in the Eastern Region of Saudi Arabia, pursuing diplomas, bachelor’s, master’s, and doctorate degrees. The inclusion criteria were enrolment as a student at King Faisal University and provision of electronic informed consent prior to participation. Respondents were informed about the study objectives and the anonymous use of their data for research purposes. The respondents excluded from the study were those who did not give their consent, and those who were not enrolled at the university where the study was being conducted. A convenience sampling technique was adopted in which the questionnaire (built using Google Forms) was shared through online media among the students. Data collection was conducted between September 2025 and January 2026.

### 2.4 Development and validation of the questionnaire

The questionnaire was developed following an extensive review of the literature on knowledge, attitudes, and perceptions related to psychoactive substances, including NPS. The questionnaire was developed and administered in English and its content validity was evaluated by a panel of 10 experts comprising five academic pharmacists from Saudi universities and five practicing pharmacists working in hospital and community pharmacy settings, all with expertise in public health and substance use. The experts assessed the questionnaire for clarity, relevance, comprehensiveness, and appropriateness of the items. Revisions were made based on their feedback to improve content validity and readability. A pilot study involving 20 students was subsequently conducted to evaluate the comprehensibility and feasibility of the questionnaire. Internal consistency reliability was high, with a Cronbach’s alpha coefficient of 0.93.

### 2.5 Instrument structure

The survey consisted of four major parts:

1. Sociodemographic Variables: This instrument included data on gender, age, education, experience with studying abroad, and prior education on NPS awareness.
2. The Knowledge Dimension: It analyzed the knowledge of respondents on various aspects of NPS, such as definition and characteristics, types of NPS, similarities of NPS to other controlled substances, possible health risks like heart disease, nervous system disorders, infections, and systemic problems. Answers were measured using multiple-choice questions and multiple-response questions.
3. The Attitude Dimension: It assessed respondents’ beliefs and perception regarding NPS use including perceived social acceptability, perceived behavioral control over NPS consumption, perceived benefits, and beliefs regarding recovery from addiction. Responses were recorded as Correct, Incorrect, or Uncertain according to predefined criteria based on current scientific evidence and public health.
4. The Perception Dimension: We explored the perceived availability of NPS (e.g., through the internet, friends, and entertainment venues), perceived motivations for NPS use (e.g., social gatherings, weight loss, and stress reduction), and personal beliefs about behavioral control over NPS consumption.

### 2.6 Ethical considerations

Participation in this study was completely voluntary. Data collection took place after obtaining electronic informed consent from all respondents. To ensure that the privacy of the respondents was upheld, no personal identifiers such as names, student IDs, or IP address were obtained. All procedures used during the study were done in accordance with the ethical principles of the Declaration of Helsinki and its subsequent amendments (21). Moreover, ethical guidelines of both national and institutional ethics committees for conducting experiments on humans were taken into consideration. The Research Ethics Committee of the Deanship of Scientific Research at King Faisal University granted approval (reference no. KFU-REC-2026-MAR-ETHICS4051) on 12^th^ March 2025.

### 2.7 Data analysis

Data were collected using Google Forms and imported into SPSS version 29 for analysis. Frequencies and percentages were used to summarize categorical variables, whereas means and standard deviations were used for continuous variables. Knowledge items were scored by assigning one point for each correct response and zero points for incorrect or uncertain responses. The total knowledge score ranged from 0 to 6 and was converted into a percentage. Knowledge levels were classified as high (≥70%), moderate (50–69%), or poor (<50%). For logistic regression analysis, respondents with high knowledge were compared with those having poor or moderate knowledge. Beliefs and perceptions regarding NPS were assessed using four items. Responses consistent with current scientific evidence were assigned one point, whereas incorrect or uncertain responses received zero points. The total belief/perception score ranged from 0 to 4 and was used in subsequent comparative and regression analyses. The total belief/perception score ranged from 0 to 4. Respondents with a score of 4 were classified as having permissive beliefs and perceptions regarding NPS for logistic regression analysis. Previous NPS use was defined as self-reported lifetime use of NPS and was recorded as a dichotomous variable (Yes/No). Chi-square test or Fisher’s exact test was used to assess associations between respondent characteristics and knowledge levels. Differences in mean belief/perception scores between groups were examined using the independent-samples t-test and one-way analysis of variance (ANOVA). Binary logistic regression analysis was performed to identify independent predictors of high knowledge and permissive beliefs and perceptions regarding NPS. Odds ratios (ORs) with 95% confidence intervals (CIs) were reported, and statistical significance was set at p < 0.05.

## 3 Results

### 3.1 Respondents’ demographics

Our study included 300 respondents for the assessment of knowledge, attitudes, and perceptions toward NPS among King Faisal university students. Notably, most of the respondents were female (222, 74.0%), while 78 (26.0%) were male. The majority were aged 18–24 years (263, 87.7%), followed by 25–34 years (25, 8.3%) and >34 years (12, 4.0%).

Most respondents were enrolled in a bachelor’s program (232, 77.3%), whereas 53 (17.7%) held a diploma and 15 (5.0%) were pursuing or had completed a master’s/PhD. A total of 102 (34.0%) had studied abroad experience and 161 (53.7%) had received prior anti-drug education. More than half reported easy access to NPS (171, 57.0%). Among them, access was mainly through online sources (121, 70.8%), followed by entertainment venues (87, 50.9%) and friends/relatives (85,49.7%). Previous NPS use was reported by 119 (39.7%), while 181 (60.3%) had never used NPS (**Table 1**).

**Table 1:** Sociodemographic characteristics and NPS accessibility and perceived use among the study respondents (N = 300).

Variable	Group	Frequency N (%)
**Gender**	Female	222 (74.0)
	Male	78 (26.0)
**Age**	18–24 years	263 (87.7)
	25–34 years	25 (8.3)
	>34 years	12 (4.0)
**Current Academic Level**	Diploma	53 (17.7)
	Bachelor’s	232 (77.3)
	Master’s/PhD	15 (5.0)
**Study Abroad Experience**	No	198 (66.0)
	Yes	102 (34.0)
**Prior Anti-Drug Education**	No	139 (46.3)
	Yes	161 (53.7)
**Easy Access to NPS**	No	129 (43.0)
	Yes	171 (57.0)
*Sources of Easy Access (n=171)*	Among Friends/Relatives	85 (49.7)
	Entertainment Venues	87 (50.9)
	Online	121 (70.8)
**Previous NPS Use**	No	181 (60.3)
	Yes	119 (39.7)

(N) Frequency, (%) Percentages, (*) Multiple responses allowed and the percentages are based on the 171 respondents who reported easy access. Bold value denotes statistical signifcance p < 0.05.

### 3.2 Reasons for NPS uses

**Figure 1:**
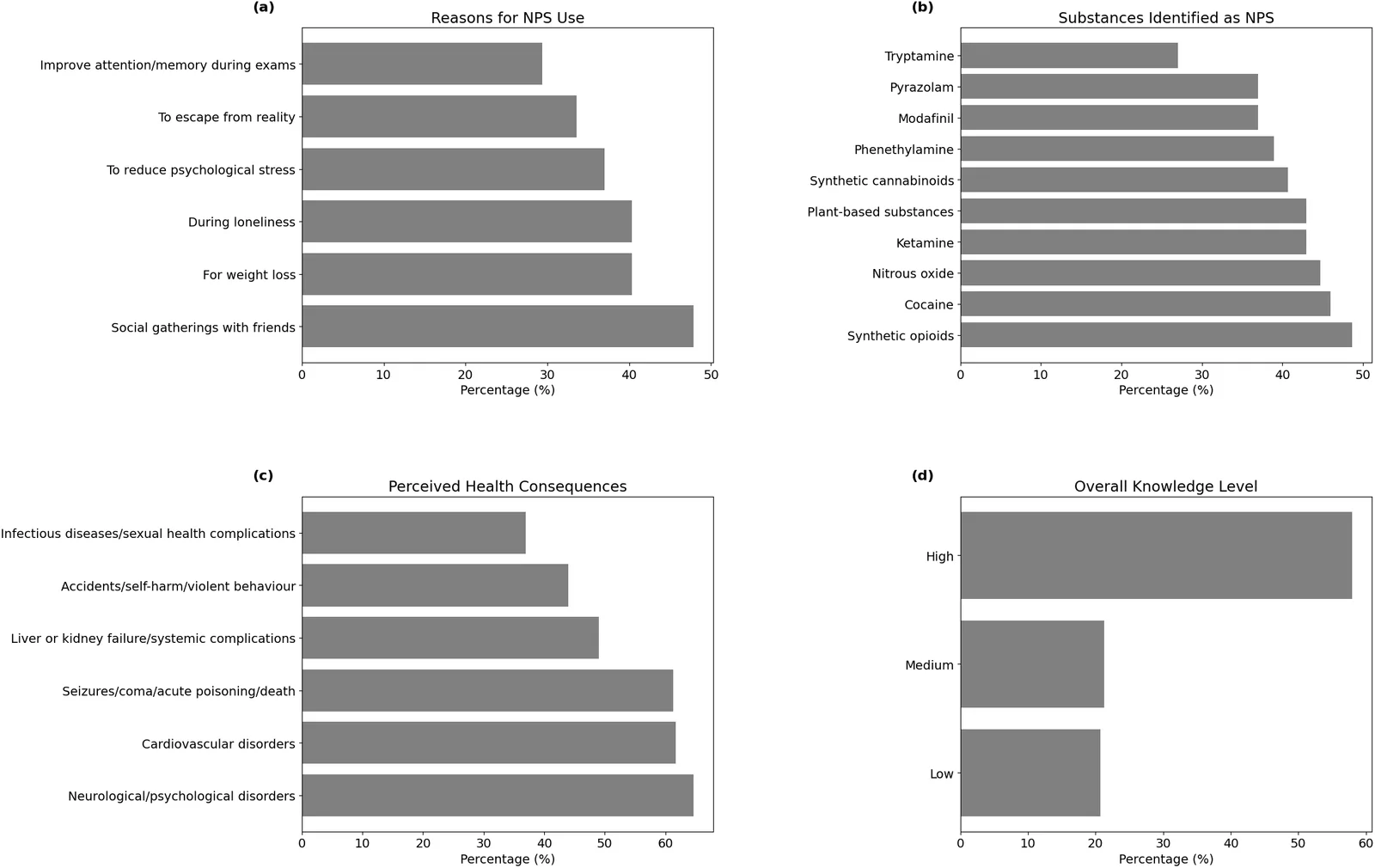
**(a)** Different reasons for NPS uses (n=119), **(b)** substances identified by respondents as NPS or NPS-related (N = 300), **(c)** perceived health consequences of NPS use (N = 300), and **(d)** overall knowledge level about NPS.

**Figure 1** shows the different reasons for using NPS among respondents who reported previous use (n=119). The most commonly reported reason was use during social gatherings with friends (47.9%), followed by use for weight loss (40.3%) and during feelings of loneliness (40.3%). A considerable proportion reported using NPS to reduce psychological stress (37.0%). Other motivations included escaping from reality (33.6%) and improving attention and memory during exams (29.4%).

### 3.3 Knowledge and attitudes towards NPS

According to **Table 2**, most respondents demonstrated moderate awareness of selected aspects of NPS. A total of 196 (65.3%) of respondents incorrectly believed that NPS represented altered forms of controlled drugs and 198 (66.0%) respondents claimed that NPS contained natural plant-based ingredients. Additionally, 189 (63.0%) respondents had some knowledge about their similar mechanisms to other psychoactive drugs and negative effects on health. Nonetheless, there was doubt among the study respondents regarding the role played by NPS in opioid use disorder, since 73 (24.3%) did not know this information and 169 (56.3%) answered the question accurately. The item related to opioid use disorder assessed awareness of claims and misconceptions concerning NPS and opioid use disorder rather than suggesting that NPS are approved or recommended for the treatment of opioid use disorder. Sexual health complications due to the use of NPS were known by only 161 (53.7%) respondents and 85 (28.3%) were unsure. Also, 164 (54.7%) respondents considered that NPS represented classical drugs.

**Table 2:** Knowledge and attitudes toward NPS among study respondents (N = 300).

Statement	Incorrect N (%)	Correct N (%)	Uncertain N (%)
Knowledge of new psychoactive substances (NPS)			
Most NPS are modified versions of controlled drugs designed to evade detection and may produce similar or stronger effects	50 (16.7)	196 (65.3)	54 (18.0)
NPS include not only chemically altered substances but also natural plant-based materials	52 (17.3)	198 (66.0)	50 (16.7)
NPS have similarities in chemical structure, mechanism of action, and biological effects to regulated drugs with comparable health risks	51 (17.0)	189 (63.0)	60 (20.0)
NPS have been suggested as a potential option for managing opioid use disorder but require further clinical validation due to health risks	58 (19.3)	169 (56.3)	73 (24.3)
Prolonged NPS use may contribute to sexual health complications	54 (18.0)	161 (53.7)	85 (28.3)
NPS are classified as drugs	68 (22.7)	164 (54.7)	68 (22.7)
Attitude and perception of new psychoactive substances (NPS)			
It is acceptable for today’s young people to try NPS once	153 (51.0)	89 (29.7)	58 (19.3)
I believe I can control the frequency and dosage of NPS use	127 (42.3)	101 (33.7)	72 (24.0)
Using NPS may increase confidence in social situations or improve performance (e.g., weight loss, energy)	143 (47.7)	106 (35.3)	51 (17.0)
Even if addicted to NPS, I can return to my pre-use state with effort and abstinence	113 (37.7)	118 (39.3)	69 (23.0)

(N) Frequency, (%) Percentages.

### 3.4 Respondents’ recognition of substances associated with NPS or NPS-related substances

**Figure 1** presents the substances that the respondents identified as NPS or NPS-related substances. Synthetic opioids were the most frequently recognized (48.7%), followed by cocaine (46.0%) and nitrous oxide (44.7%). Ketamine and plant-based substances were each identified by 43.0% of respondents. Recognition of synthetic cannabinoids was reported by 40.7%, while phenethylamine accounted for 39.0%. Lower proportions identified modafinil and pivaloyl acetate (37.0% each), and tryptamine (27.0%).

### 3.5 Perceived health consequences of NPS use

**Figure 1** shows the perceived health consequences of NPS use among respondents. Neurological and psychological disorders were the most frequently identified effects (64.7%), followed by cardiovascular disorders (61.7%) and seizures, coma, acute poisoning, or death (61.3%). Liver or kidney failure and other systemic complications were reported by 49.0%, while 44.0% recognized the risk of accidents, self-harm, and violent behavior. The least identified consequence was increased risk of infectious or sexual health complications (37.0%).

### 3.6 Association between respondent characteristics and knowledge level about NPS

**Table 3** shows that there is a significantly higher proportion of females demonstrated a high knowledge level (142, 64.0%) compared with males (32, 41.0%), while males more frequently had low to moderate knowledge (46, 59.0%) than females (80, 36.0%) (χ² = 12.467, p < 0.001). Knowledge level did not differ significantly across age groups (p = 0.799), although a high level was observed in 151 (57.4%) of those aged 18–24 years, 15 (60.0%) of those aged 25–34 years, and 8 (66.7%) of those aged >34 years. Similarly, academic level (p = 0.105), study abroad experience (p = 0.304), prior anti-drug education (p = 0.704), easy access to NPS (p = 0.107), and previous NPS use (p = 0.153) showed no significant association with knowledge.

**Table 3:** Association between respondents’ characteristics and knowledge level about NPS (N = 300).

Variable	Low to moderate knowledge n (%)	High knowledge n (%)	Test value	Significance (p)
**Gender**			12.467	**<0.001^a^**
Female	80 (36.0)	142 (64.0)		
Male	46 (59.0)	32 (41.0)		
**Age**			0.448	0.799^b^
18–24 years	112 (42.6)	151 (57.4)		
25–34 years	10 (40.0)	15 (60.0)		
>34 years	4 (33.3)	8 (66.7)		
**Current academic level**			4.505	0.105^a^
Diploma	29 (54.7)	24 (45.3)		
Bachelor’s	92 (39.7)	140 (60.3)		
Master’s/PhD	5 (33.3)	10 (66.7)		
**Study abroad experience**			1.055	0.304^a^
No	79 (39.9)	119 (60.1)		
Yes	47 (46.1)	55 (53.9)		
**Prior anti-drug education**			0.144	0.704^a^
No	60 (43.2)	79 (56.8)		
Yes	66 (41.0)	95 (59.0)		
**Easy access to NPS**			2.597	0.107^a^
No	61 (47.3)	68 (52.7)		
Yes	65 (38.0)	106 (62.0)		
**Previous NPS use**			2.045	0.153^a^
No	82 (45.3)	99 (54.7)		
Yes	44 (37.0)	75 (63.0)		

(a) Chi-Square Test, (b) Fisher’s Exact Test. Bold value denotes statistical signifcance p < 0.05.

**Table 4** shows the results of the binary logistic regression analysis for predictors of high knowledge and awareness of NPS. Gender was the only variable significantly associated with higher knowledge. Male respondents had significantly lower odds of having high knowledge compared with females (AOR = 0.407, 95% CI: 0.229–0.722, p = 0.002). Age was not a significant predictor (AOR = 1.286, 95% CI: 0.746–2.217, p = 0.365). Other factors like education and experience of NPS was not associated significantly.

**Table 4:** Binary logistic regression analysis for predictors of high knowledge and awareness.

Variable	B	S.E.	Sig.	Exp(B)	95% CI for Exp(B)
Gender (Male)	-0.900	0.293	**0.002**	0.407	0.229 – 0.722
Age	0.252	0.278	0.365	1.286	0.746 – 2.217
Higher Education	0.366	0.276	0.185	1.442	0.839 – 2.477
Study Abroad Experience (Yes)	-0.280	0.289	0.332	0.756	0.429 – 1.332
Prior Anti-Drug Education (Yes)	0.141	0.254	0.578	1.151	0.700 – 1.893
Easy Access to NPS (Yes)	0.114	0.312	0.716	1.120	0.607 – 2.067
Previous NPS Use (Yes)	0.458	0.342	0.181	1.581	0.809 – 3.089
Constant	-0.267	0.430	0.535	0.766	—

Bold value denotes statistical signifcance p < 0.05.

### 3.7 Respondents’ aggregate knowledge level of NPS

**Figure 1** shows the overall knowledge level regarding NPS among respondents. More than half of the respondents demonstrated a high level of knowledge (58.0%), while more than one-fifth had medium knowledge (21.3%). A similar proportion showed low knowledge (20.7%).

### 3.8 Comparison of permissive belief scores across respondent subgroups

**Table 5** shows the differences in attitude scores according to respondents’ characteristics. Females had significantly higher attitude scores than males (1.52 ± 1.45 vs 0.97 ± 1.25; F = 8.830, p = 0.003). Attitude scores did not differ significantly across age groups (p = 0.382) or academic level (p = 0.320). Although respondents with study abroad experience had slightly higher mean scores than those without (1.54 ± 1.54 vs 1.30 ± 1.35), the difference was not statistically significant (p = 0.164). Similarly, prior anti-drug education showed no significant effect on attitude (p = 0.924). In contrast, respondents who reported easy access to NPS had markedly higher attitude scores than those without access (1.79 ± 1.46 vs 0.84 ± 1.17; F = 37.071, p < 0.001). Previous NPS users also demonstrated significantly higher attitude scores compared to non-users (2.00 ± 1.48 vs 0.97 ± 1.22; F = 42.884, p < 0.001).

**Table 5:** Differences in permissive belief scores according to respondents’ characteristics (N = 300).

Variable	Group	Mean ± SD	F	Sig.
**Gender**	Female	1.52 ± 1.45	8.830	**0.003 *^a^***
	Male	0.97 ± 1.25		
**Age**	18–24 years	1.42 ± 1.45	0.964	0.382 ***^b^***
	25–34 years	1.12 ± 1.20		
	>34 years	1.00 ± 1.21		
**Current Academic Level**	Diploma	1.11 ± 1.14	1.143	0.320 ***^b^***
	Bachelor’s	1.44 ± 1.49		
	Master’s/PhD	1.40 ± 1.12		
**Study Abroad Experience**	No	1.30 ± 1.35	1.950	0.164 ***^a^***
	Yes	1.54 ± 1.54		
**Prior Anti-Drug Education**	No	1.39 ± 1.41	0.009	0.924 ***^a^***
	Yes	1.37 ± 1.44		
**Easy Access to NPS**	No	0.84 ± 1.17	37.071	**<0.001 *^a^***
	Yes	1.79 ± 1.46		
**Previous NPS Use**	No	0.97 ± 1.22	42.884	**<0.001 *^a^***
	Yes	2.00 ± 1.48		

(a) Independent T Test, (b) ANOVA. Bold value denotes statistical signifcance p < 0.05.

### 3.9 Logistic regression analysis of variables associated with permissive beliefs and perceptions regarding NPS

**Table 6** shows the binary logistic regression analysis for predictors of permissive beliefs and perception toward NPS relates social acceptability. Male respondents had significantly lower odds of having a permissive belief compared with females (AOR = 0.537, 95% CI: 0.295–0.979, p = 0.042). Respondents who reported easy access to NPS had more than twice the odds of a permissive beliefs (AOR = 2.298, 95% CI: 1.233–4.285, p = 0.009). Similarly, previous NPS users were significantly more likely to have a permissive belief compared with non-users (AOR = 2.502, 95% CI: 1.209–5.176, p = 0.013).

**Table 6:** Binary logistic regression analysis for predictors of permissive beliefs and perception of NPS.

Variable	B	S.E.	Sig.	Exp(B)	95% CI for Exp(B)
Gender (Male)	-0.621	0.306	**0.042**	0.537	0.295 – 0.979
Age	0.026	0.280	0.927	1.026	0.593 – 1.775
Higher Education	0.073	0.291	0.801	1.076	0.609 – 1.902
Study Abroad Experience (Yes)	-0.156	0.309	0.613	0.855	0.466 – 1.568
Prior Anti-Drug Education (Yes)	-0.346	0.268	0.196	0.707	0.418 – 1.195
Easy Access to NPS (Yes)	0.832	0.318	**0.009**	2.298	1.233 – 4.285
Previous NPS Use (Yes)	0.917	0.371	**0.013**	2.502	1.209 – 5.176
Constant	0.033	0.436	0.941	1.033	—

Bold value denotes statistical signifcance p < 0.05.

## 4 Discussion

### 4.1 Knowledge and awareness of NPS

Findings from this cross-sectional study illustrate that although 58% of the respondents showed good knowledge about NPS, there remain important gaps and concerning behavior patterns persist. It is worth mentioning that 57% of respondents indicated having easily available NPS, mostly through Internet platforms (70.8% among users), and 39.7% reported ever using NPS, making them a serious public health problem in Saudi universities. Women had significantly higher knowledge scores compared to men (64.0% versus 41.0%, p <0.001), and being male was found to be an independent predictor of low knowledge (AOR = 0.407, 95% CI: 0.229–0.722, p = 0.002). This result is at odds with the situation observed among Serbians, where women were found to know 36.5% fewer abbreviations for new psychoactive substances (IRR = 0.635; p = 0.049) (14), which implies that differences between genders regarding knowledge about NPS can be culturally specific and might indicate differences in sensitivity towards health communications or information-seeking behavior among societies. Culturally targeted health communication strategies have been found to exhibit gender differences, with the use of culturally tailored and loss-framed messages having stronger impacts on men (22).

Awareness was generally high, significant ambiguity existed regarding sexual health problems (28.3%) and NPS’s role in opioid use disorder (24.3%). One concern tied to NPS risks involves rising cases of pharming - using prescription or over-the-counter drugs without medical need (5). Because such medicines are easy to obtain, people might treat them as harmless. Young adults can overlook serious consequences due to false assumptions about safety (5). In addition, the ambiguity is consistent with the results of general population studies showing that there is still very little recognition about NPS-induced sexual problems such as erectile dysfunction and loss of libido (23, 24). In addition, lower awareness regarding the dangers of NPS and sexual infections (37%), despite their links to NPS use, is an educational gap that needs to be addressed, especially because there are already established links between the former and risky activities (25).

Moreover, the belief that synthetic cathinones and novel opioids are safer substitutes for heroin or prescription opioids is a misconception that has been recorded by both people who use drugs and medical professionals, resulting in an underestimation of their impact on the aggravation of opioid use disorder and overdose risk (26). These persistent deficiencies in knowledge suggest that existing educational programs on drugs do not effectively incorporate the specific features of NPS such as chemical variability, unknown toxicity, and legal uncertainty. This calls for an innovative, evidence-based approach to educating about NPS. From research on health information seeking, it is known that men and women have varied motivational determinants, with men being more affected by subjective norms and women by perceived seeking control (27). Hence, gender-specific communication approaches are crucial when conducting outreach efforts.

### 4.2 Attitudes towards NPS

Attitudinal factors were also concerning. Previous use of NPS (AOR = 2.502; 95% CI: 1.209-5.176, p = 0.013) and accessibility (AOR = 2.298; 95% CI: 1.233-4.285, p = 0.009) were found to positively influence attitudes towards NPS. A total of 42.3% thought they could control the dosage and frequency, and 35.3% felt there were performance-enhancing effects. “Illusion of control” fits well with the observation that knowledge is minimally associated with safe behavior (r = –0.05), which may be attributed to psychological reactance or cognitive dissonance reduction amongst experienced individuals (28). The social environment had an important effect on usage frequency, with 47.9% having consumed it when among their peers while 40.3% took NPS for purposes of losing weight. The respondents correctly identified health hazards such as neurotoxicity (64.7%) and cardiotoxicity (61.7%), which have been reported for NPS with serious consequences up to death (15, 16). However, links between NPS use and severe mental health issues - like psychosis, suicide, or self-harm- require stronger attention in educational programs (9, 10). When symptoms appear after using NPS, doctors must take notice; such drugs may spark or worsen psychological problems, even in people who have never shown signs of psychological problems before (9).

### 4.3 Access to NPS

Access to NPS was easy, mostly through websites (70.8%). The prevalence of the use of online websites for obtaining NPS is reflected in European multi-country figures on the use of the internet market as the main source for NPS acquisition, along with the adoption of effective marketing tactics to entice younger buyers (29, 30). All these results clearly indicate that although Saudi students are aware of basic knowledge about NPS, their knowledge does not translate into behavior. The accessibility of substances by means of technology platforms poses a huge challenge for preventive programs. Online platforms dominated access to NPS in our cohort. However, patterns of substance misuse differ across regions. European research indicates that the non-medical use of over-the-counter and prescription medications—a phenomenon known as ‘pharming’—represents a significant public health concern (6, 7). A recent Italian study examined over-the-counter drug misuse in the general population and found that misuse was associated with knowledge of OTC effects, while higher education was protective, particularly among younger individuals (8). In contrast, our Saudi university sample demonstrated higher NPS-specific awareness, with nearly 60% showing a high level of knowledge of NPS risks. Yet, permissive attitudes toward casual use remained common in both populations. The Italian findings highlighted that respondents often trusted medication labels and perceived pharmaceutical products as low risk, whereas our results emphasized the role of digital accessibility for synthetic substances rather than confidence in packaging. These cross-cultural contrasts underscore that one-size-fits-all educational interventions may prove ineffective without tailoring to region-specific substance availability and access patterns.

### 4.4 Limitations of the study

Some limitations should be considered. Firstly, the cross-sectional nature of the study does not allow for causality to be established, and even with anonymity, self-reports are always prone to social desirability and memory bias. Secondly, the convenience sample conducted in only one institution in the Eastern Province means that the results cannot be generalized to the whole of Saudi Arabia due to its wide range of regions and diversity. Thirdly, although validated by experts, the questionnaire was not subject to psychometric testing, and without biological testing, it is likely that the reported rate of NPS use is underreported. In addition, the attitude and perception items were assessed using a Correct/Incorrect/Uncertain response format rather than a Likert-type scale. Therefore, the findings should be interpreted as reflecting respondents’ beliefs and perceptions regarding NPS rather than psychometrically validated attitudinal constructs. Nonetheless, there were several strengths of the current study, which included being one of the first studies in Saudi Arabia on NPS knowledge, attitudes, and perceptions among university students that fills an important literature gap since previous studies had covered conventional drugs.

There are several policy implications that come directly from the findings reported in this study. Any educational efforts to curb the prevalence of NPS need to focus on their unique attributes and not be generally framed around anti-drug campaigns, with the possibility of gender-oriented programs increasing effectiveness. Considering the importance of online drug purchases, there is a need for increased surveillance on the internet as well as coordination between regulatory agencies and various platforms where such drugs can be procured online. Universities need to address the issues driving the use of NPS among students, especially body weight management and stress relief, by providing evidence-based alternative options.

## 5 Conclusions

This cross-sectional study suggests that despite relatively high overall awareness concerning NPS, there are considerable gaps in knowledge regarding the particular harms associated with NPS use and the categorization of such substances. Ease of access, particularly online, along with past use were found to be independent predictors of favorable attitudes towards NPS, while awareness on its own could not prevent permissive attitudes from developing. Additionally, gender differences were apparent in this study, showing that females are better aware of NPS and are more cautious than males. The results obtained suggest that conventional approaches to drug use education are insufficient to address the problem of NPS, requiring the development of gender-specific strategies to be applied on campuses. The increased regulation of NPS sales online and the inclusion of relevant information in health programs of universities should be prioritized, since they are crucial for addressing the current problem in accordance with Quality of Life goals outlined in the Saudi Vision 2030. Further longitudinal studies on the topic, utilizing multiple institutions, are recommended to achieve a more detailed understanding of the issue.

## Data Availability

The original contributions presented in the study are included in the article/**Supplementary Material**. Further inquiries can be directed to the corresponding author.

## References

[1] The European Union Drugs Agency (EUDA) . EU Drug Market: New Psychoactive Substances - Global Context (2024). Available online at: https://www.euda.europa.eu/publications/eu-drug-markets/new-psychoactive-substances/global-context_sv (Accessed April 21, 2026).

[2] United Nations: Office on Drugs and Crime . World Drug Report (2021). Available online at: https://www.unodc.org/unodc/en/data-and-analysis/wdr2021.html (Accessed April 21, 2026).

[3] Shafi A Berry AJ Sumnall H Wood DM Tracy DK . New psychoactive substances: a review and updates. Ther Adv Psychopharmacol. (2020) 10. doi: 10.1177/2045125320967197 33414905 PMC7750892

[4] Miliano C Margiani G Fattore L De Luca M . Sales and advertising channels of new psychoactive substances (NPS): Internet, social networks, and smartphone apps. Brain Sci. (2018) 8:123. doi: 10.3390/brainsci8070123 29966280 PMC6071095

[5] Chiappini S Schifano F . What about “Pharming”? Issues regarding the misuse of prescription and over-the-counter drugs. Brain Sci. (2020) 10:736. doi: 10.3390/brainsci10100736 33066476 PMC7602178

[6] Schifano F Chiappini S Corkery J Guirguis A . Abuse of prescription drugs in the context of novel psychoactive substances (NPS): A systematic review. Brain Sci. (2018) 8:73. doi: 10.3390/brainsci8040073 29690558 PMC5924409

[7] Schifano F Chiappini S Miuli A Mosca A Santovito MC Corkery JM . Focus on over-the-counter drugs’ misuse: A systematic review on antihistamines, cough medicines, and decongestants. Front Psychiatry. (2021) 12. doi: 10.3389/fpsyt.2021.657397 34025478 PMC8138162

[8] Chiappini S Ceci F Mosca A Di Carlo F Burkauskas J Pettorruso M . Knowledge and use of over-the-counter drugs in Italy: An exploratory survey-based study in the general population. Curr Neuropharmacol. (2023) 21:133–41. doi: 10.2174/1570159X20666220714104231 35838215 PMC10193759

[9] Mosca A Mancusi G Chiappini S Miuli A Cavallotto C Corkery JM . Novel psychoactive substances (NPS) as a risk factor for psychosis: A systematic review of the literature. Neurosci Biobehav Rev. (2025) 179:106431. doi: 10.1016/j.neubiorev.2025.106431 41120031

[10] Chiappini S Mosca A Miuli A Santovito MC Orsolini L Corkery JM . New psychoactive substances and suicidality: A systematic review of the current literature. Med (B Aires). (2021) 57:580. doi: 10.3390/medicina57060580 34204131 PMC8226910

[11] Saudi Vision 2030 - Quality of Life Program (2024). Available online at: https://www.vision2030.gov.sa/en/explore/programs/quality-of-life-program (Accessed April 21, 2026).

[12] Radwan R . How Saudi Arabia prioritizes rehabilitation in its crackdown on meth, Captagon and other narcotics. In: Arab News Japan. Tokyo: Name Arab News. (2023). Available online at: https://www.arabnews.jp/en/saudi-arabia/article_98158/ (Accessed April 21, 2026).

[13] Al-Haqwi AI . Perception among medical students in Riyadh, Saudi Arabia, regarding alcohol and substance abuse in the community: a cross-sectional survey. Subst Abuse Treat Prev Policy. (2010) 5:2. doi: 10.1186/1747-597X-5-2 20092658 PMC2832638

[14] Mijatović Jovin V Skoko N Tomas A Živanović D Sazdanić D Gvozdenović N . New psychoactive substances: Awareness and attitudes of future health care professionals in Serbia. Int J Environ Res Public Health. (2022) 19:14877. doi: 10.3390/ijerph192214877 36429596 PMC9691219

[15] Corkery JM Schifano F Martinotti G . How deaths can help clinicians and policy‐makers understand the risks of novel psychoactive substances. Br J Clin Pharmacol. (2020) 86:482–98. doi: 10.1111/bcp.14183 31770457 PMC7080619

[16] Ugan Atik S Dedeoglu R Varol F Cam H Eroglu AG Saltik L . Cardiovascular side effects related with use of “Bonzai”: Two case reports. Turk Pediatri Ars. (2015) 50:61–4. doi: 10.5152/tpa.2015.2609 26078698 PMC4462325

[17] Molloy BK Stock ML Dodge T Aspelund JG . Predicting future academic willingness, intentions, and nonmedical prescription stimulant (NPS) use with the theory of reasoned action and prototype/willingness model. Subst Use Misuse. (2019) 54:2251–63. doi: 10.1080/10826084.2019.1645175 31359819

[18] Syed W Iqbal A Siddiqui NA Mothana RA Noman O . Attitudes and associated demographic factors contributing towards the abuse of illicit drugs: A cross-sectional study from health care students in Saudi Arabia. Med (B Aires). (2022) 58:322. doi: 10.3390/medicina58020322 35208645 PMC8878882

[19] Alshehri FS Ashour AM Bafhaid HS Algarni AS Harbi MH Alorfi NM . University students’ knowledge and attitudes toward substance abuse: A cross-sectional study from Saudi Arabia. Healthcare. (2025) 13:1122. doi: 10.3390/healthcare13101122 40427959 PMC12110973

[20] Kfoury R Salameh P Peyriere H . From knowledge to attitude: design and initial validation of scales for assessing psychoactive substance consumption among university students. Front Public Health. (2025) 13. doi: 10.3389/fpubh.2025.1713133 41477228 PMC12747911

[21] The World Medical Association . WMA - the World Medical Association-Declaration of Helsinki (2008). Available online at: https://www.wma.net/what-we-do/medical-ethics/declaration-of-helsinki/doh-oct2008/ (Accessed April 21, 2026).

[22] Lucas T Hayman LW Blessman JE Asabigi K Novak JM . Gain versus loss‐framed messaging and colorectal cancer screening among African Americans: A preliminary examination of perceived racism and culturally targeted dual messaging. Br J Health Psychol. (2016) 21:249–67. doi: 10.1111/bjhp.12160 26333060

[23] Benford DM Caplan JP . Psychiatric sequelae of Spice, K2, and synthetic cannabinoid receptor agonists. Psychosomatics. (2011) 52:295. doi: 10.1016/j.psym.2011.01.004 21565605

[24] Novel Psychoactive Treatment UK Network (NEPTUNE) . Guidance on the Clinical Management of Acute and Chronic Harms of Club Drugs and Novel Psychoactive Substances (2015). Available online at: https://ndews.umd.edu/sites/ndews.umd.edu/files/pubs/UK10_NEPTUNE%20NPS%20guidance%20(2015).pdf.

[25] Weinstein AM Rosca P Fattore L London ED . Synthetic cathinone and cannabinoid designer drugs pose a major risk for public health. Front Psychiatry. (2017) 8. doi: 10.3389/fpsyt.2017.00156 28878698 PMC5572353

[26] Palamar JJ Acosta P . A qualitative descriptive analysis of effects of psychedelic phenethylamines and tryptamines. Hum Psychopharmacol - Clin Exp. (2020) 35. doi: 10.1002/hup.2719 31909513 PMC6995261

[27] Bidmon S Terlutter R . Gender differences in searching for health information on the internet and the virtual patient-physician relationship in Germany: Exploratory results on how men and women differ and why. J Med Internet Res. (2015) 17:e156. doi: 10.2196/jmir.4127 26099325 PMC4526954

[28] Wang X Zhang L . Perception, attitude and abuse intention about new psychoactive substances and their associates among university students in Shanghai. Chin J Public Health. Chin J Public Health. (2022) 38. doi: 10.11847/zgggws1135911

[29] Corazza O Valeriani G Bersani FS Corkery J Martinotti G Bersani G . Spice,” “Kryptonite,” “Black Mamba”: An overview of brand names and marketing strategies of novel psychoactive substances on the web. J Psychoactive Drugs. (2014) 46:287–94. doi: 10.1080/02791072.2014.944291 25188698

[30] Werse B Benschop A Kamphausen G van Hout M-C Henriques S Silva JP . Sharing, group-buying, social supply, offline and online dealers: how users in a sample from six European countries procure new psychoactive substances (NPS). Int J Ment Health Addict. (2019) 17:1237–51. doi: 10.1007/s11469-018-0043-1 30311153

