# Supplementary material for: Knowledge, attitudes, and perception toward new psychoactive substances among Saudi university students: a cross-sectional study
Source: Front Psychiatry. 2026 Jul 8;17:1877711. doi: 10.3389/fpsyt.2026.1877711 (PMC13388320; doi:10.3389/fpsyt.2026.1877711)
Supplement: Supplementary file 1 [file Table1.docx]

**Study Title：Investigation of University Students’Knowledge, Attitudes, and Practices(KAP) Regarding Novel Psychoactive Substances(NPS)**

**Purpose**
This research investigates students' awareness, attitudes, and behaviors regarding the addictive risks and health impacts of new psychoactive substances (NPS). Your participation will contribute to developing effective campus prevention strategies.

**Key Information**

☑ (Anonymity): No personal identifiers (name/student ID/IP) are collected
☑ (Confidentiality): Data stored on encrypted servers for 3 years, accessible only to the research team
☑ (Voluntary): You may withdraw anytime without penalty

**Ethical Compliance**Approved by the Fujian Police College Ethics Committee (The ethics number is EC-20230824-1022), in accordance with Declaration of Helsinki.

**By proceeding, you confirm:**
☐ 1. Current enrollment in a degree program (associate/bachelor’s/master’s)
☐ 2. If under 16: Guardian consent has been obtained and submitted
☐ 3. Consent to anonymous data use for academic purposes

**Section1: Participant Information**

1. Gender:

☐ Male

☐ Female

2. Age: ______ years

3. Ethnicity:

☐ Han

☐ Ethnic minority (specify: ______)

4. Current academic level:

☐ Associate degree

☐ Bachelor’s degree

☐ Master’s degree

☐ Doctoral degree

5. Field of study:

______

*(Options: Philosophy, Economics, Law, Education, Literature, History, Science, Engineering, Agriculture, Medicine, Military Science, Management, Arts, Other: ______)*

6. Study abroad experience:

☐ Yes

☐ No

7. Relationship status:

☐ Single

☐ In a relationship

☐ Married

8. Residence type:

☐ Urban

☐ Rural

9. Monthly household income:

☐ <8,000 RMB

☐ 8,000–12,000 RMB

☐ 12,000–20,000 RMB

☐ >20,000 RMB

10. Prior anti-drug education:

☐ Yes

☐ No

**Section2: Knowledge Dimension**

1. Most new psychoactive substances (NPS) are modified versions of controlled chemicals (drugs) created by illicit individuals to evade detection, resulting in substances with effects similar or more potent than regulated drugs.

☐correct ☐ incorrect ☐ uncertain

1. NPS includes not only chemically altered substances but also natural plant-based materials.

☐correct ☐ incorrect ☐ uncertain

1. China has implemented "pre-control" measures on certain NPS, considering them as the third generation of drugs following traditional drugs and synthetic drugs.

☐correct ☐ incorrect ☐ uncertain

NPS includes: ☐correct ☐ incorrect ☐ uncertain

1. Nitrous Oxide (Laughing Gas)
2. Ketamine-related substances (Bath Salts, Weed Killer, Jelly)
3. Ketamine (Special K, Coca-Cola, Fairy Water)
4. Synthetic Cannabinoids (Bouncing Candy, Little Branches, Natasha, E-cigarette Oil)
5. Ecstasy-like substances
6. Synthetic Opioids (e.g., Fentanyl)
7. New Benzodiazepine-like drugs (e.g., Zolpidem, Dichloroisopropyl)
8. Cocaine
9. Phenethylamine (Psychedelic Stamps)
10. Tryptamine (Number Zero Capsules, Firefox, G-spot Liquid)
11. Fentanyl analogs
12. Plant-based substances (Chat Grass, Kratom Leaves, Salvia)
13. Modafinil, Pivaloylacetate (Smart Drugs)

5.NPS possess similarities in chemical structures, mechanisms of action, and biological effects to regulated drugs, which may lead to comparable health risks.

☐correct ☐ incorrect ☐ uncertain

6.Abusing NPS can lead to addiction, withdrawal reactions, harm to personal health, and societal risks, exhibiting both addictive and toxic characteristics.

☐correct ☐ incorrect ☐ uncertain

7.Abusing NPS may cause: ☐correct ☐ incorrect ☐ uncertain

(1) Accelerated heart rate, increased blood pressure

(2) Impaired judgment

(3) Blurred consciousness and mental disorders

(4) Induction of accidents, self-harm, and violent behavior

(5) Seizures, coma, shock, or even death

(6) Increased risk of cardiovascular diseases

(7) Elevated cancer risk

(8) Acute poisoning

(9) Liver and kidney function failure

(10) Mania, paranoia, and persecutory delusions

8.The use of NPS has been suggested as a potential method for managing opioid use disorder (OUD), though significant health risks may occur during this process and requires further clinical validation

☐correct ☐ incorrect ☐ uncertain

9. NPS use may increase the risk of HIV infection.

☐correct ☐ incorrect ☐ uncertain

10.Prolonged NPS use may contribute to sexual health complications.

☐correct ☐ incorrect ☐ uncertain

**Section3:Attitude Dimension**

1.I think it is acceptable for today's young people to try NPS once.

☐strongly agree ☐ agree ☐ neutral ☐disagree ☐ strongly disagree

2. I believe I can control the frequency and dosage of NPS use.

☐strongly agree ☐ agree ☐ neutral ☐disagree ☐ strongly disagree

3.I think using NPS can enhance the atmosphere of parties or increase popularity at gatherings.

☐strongly agree ☐ agree ☐ neutral ☐disagree ☐ strongly disagree

4.I believe using NPS can boost my confidence in social situations.

☐strongly agree ☐ agree ☐ neutral ☐disagree ☐ strongly disagree

5.I think occasional use of NPS can enhance sexual performance.

☐strongly agree ☐ agree ☐ neutral ☐disagree ☐ strongly disagree

6.I believe using a small amount of NPS can help with weight loss.

☐strongly agree ☐ agree ☐ neutral ☐disagree ☐ strongly disagree

7.I think using NPS will make me feel energetic.

☐strongly agree ☐ agree ☐ neutral ☐disagree ☐ strongly disagree

8.I believe using NPS for entertainment and leisure purposes is acceptable.

☐strongly agree ☐ agree ☐ neutral ☐disagree ☐ strongly disagree

9.I believe that even if I become addicted or dependent on a certain NPS, with effort and abstinence for a period, I can return to the state before using NPS.

☐strongly agree ☐ agree ☐ neutral ☐disagree ☐ strongly disagree

1. NPSs are classified as drugs.

☐strongly agree ☐ agree ☐ neutral ☐disagree ☐ strongly disagree

**Section4: Practice Dimension**

I can easily access NPS in __. ☐always ☐ often ☐ sometimes ☐occasionally ☐never

1. Entertainment venues

(2) Online

(3) Among friends/relatives

2. I have used NPS at social gatherings with friends.

☐always ☐ often ☐ sometimes ☐occasionally ☐ never

3. I have used NPS when alone.

☐always ☐ often ☐ sometimes ☐occasionally ☐ never

4. I have used NPS during sexual activities.

☐always ☐ often ☐ sometimes ☐occasionally ☐ never

5. I have used a specific NPS to enhance my attention and memory for important exams.

☐always ☐ often ☐ sometimes ☐occasionally ☐ never

6. I have used a certain NPS for weight loss.

☐always ☐ often ☐ sometimes ☐occasionally ☐ never

7. I have used a specific NPS to escape reality or reduce life stress.

☐always ☐ often ☐ sometimes ☐occasionally ☐ never

1. I have used a specific NPS to break free from limited thinking and enhance creativity.

☐always ☐ often ☐ sometimes ☐occasionally ☐ never
